# Characterization of exosomes derived from IPEC-J2 treated with probiotic *Bacillus amyloliquefaciens* SC06 and its regulation of macrophage functions

**DOI:** 10.3389/fimmu.2022.1033471

**Published:** 2022-11-09

**Authors:** Xiaogang Xu, Rongrong Liu, Xuqiang Zhou, Zhongshan Zhang, Tianjun Zhu, Yingying Huang, Lan Chai, Yazhen Wang, Zhenlei Zhao, Weifen Li, Genxiang Mao

**Affiliations:** ^1^ Geriatrics Institute of Zhejiang Province, Department of Geriatrics, Affiliated Zhejiang Hospital, Zhejiang University School of Medicine, Zhejiang University, Hangzhou, China; ^2^ Key Laboratory of Molecular Animal Nutrition of the Ministry of Education, Institute of Feed Science, College of Animal Sciences, Zhejiang University, Hangzhou, China; ^3^ College of Life Science, Zhejiang Chinese Medical University, Hangzhou, China; ^4^ Key Laboratory of Vector Biology and Pathogen Control of Zhejiang Province, Huzhou University, Huzhou, China; ^5^ Core Facilities, School of Medicine, Zhejiang University, Hangzhou, China

**Keywords:** probiotics, *Bacillus amyloliquefaciens* SC06, exosomes, *in vitro*, macrophages, IPEC-J2

## Abstract

Probiotics can maintain or improve health by modulating the response of immune cells in the gastrointestinal tract. However, the mechanisms by which probiotics promote macrophage (Mφ) activity are poorly understood. Here, we evaluated exosomes derived from intestinal epithelial cells treated with *Bacillus amyloliquefaciens* SC06 (Ba) and investigated the regulation of Mφ phagocytosis, apoptosis, and polarization. We isolated two exosomes from intestinal porcine epithelial cell lines (IPEC-J2) with or without Ba-treatment, named Ba-Exo and Exo, respectively. They had typical sizes and a cup-shaped morphology, and their surfaces presented typical exosomes-associated proteins, including CD63, ALIX, and TSG101. Ba-Exo and Exo could entrer Mφ (3D4/21 cells) effectively. Moreover, an *in vitro* phagocytosis assay demonstrated that Ba-Exo can promote phagocytosis of Mφ. Similar to Exo, Ba-Exo had no effect on Mφ apoptosis. Furthermore, Ba-Exo significantly increased inducible nitric oxide synthase (iNOS), declined the expression of arginase 1 (Arg1) in Mφ, and stimulated Mφ polarization to M1. To explore the differences in the regulation of Mφ polarization between Ba-Exo and Exo, we performed reverse transcription quantitative polymerase chain reaction analysis of the small RNAs and found that miR-222 increased in the Ba-Exo group compared to that in the Exo group. These results provide a new perspective on the relationship between probiotics and intestinal immunity.

## Introduction

Macrophages (Mφ), which are derived from blood mononuclear cells, are important antigen-presenting cells of the intestinal immune system. Mφ can not only ingest and eliminate pathogens, but also activate the immune inflammatory response, providing a connection between innate and adaptive immunity (1). Recent studies have found that Mφ polarization plays an important role in the removal of pathogenic bacteria during the intestinal immune process while controlling moderate inflammatory responses and maintaining body health and immune homeostasis. Local micro-environmental stimuli can trigger polarization of Mφ: M1 and M2 macrophage (2, 3). Probiotics play a role in defining and maintaining a delicate balance between the necessary and excessive defense mechanisms including innate and adaptive immune responses (4). M1 macrophages expresses inducible nitric oxide synthase (iNOS), which synthesizes and accumulates reactive oxygen species and nitric oxide, and secretes pro-inflammatory cytokines (such as interleukin (IL)-12, tumor necrosis factor-α and IL-1β), lysozyme and antimicrobial peptides. They mainly resist and eliminate foreign antigens and can 1) present antigens timely manner, 2) activate T and B lymphocytes, 3) promote B cells to differentiate into plasma cells, and 4) generate corresponding antibody responses (5). M2 macrophages can be divided into three subgroups: M2a, M2b, and M2c. M2a cells highly expresse arginase 1 (Arg1), Ym1, Fizz1, mannose receptor and scavenger receptor, CD163, which are involved in tissue repair. Mφ interact with microbiomes that develop mutualistic relationships with the host (6). Microbes are recognized by pattern recognition receptors, the transcribed response of the host, and secreted effector molecules including metabolites and antigens, to transfer signals to stimulate Mφ (7).

Probiotics are live microorganisms that confer health benefits to the host when administered in adequate amounts (8). Accumulating evidence suggests that probiotics can also induce Mφ polarization. Probiotics can promote intestinal health by improving animal growth performance through non-immune and immunomodulatory pathways (9). *Lactobacillus brevis* G-101, isolated from kimchi, induces M2 macrophages by inhibiting the IRAK1/NF-κB, MAPK and AKT signaling pathways and alleviates intestinal inflammation in a mouse model of enteritis (10). *Lactobacillus plantarum* CLP-0611 was orally administered to increase the expression of the M2 typical marker molecules IL-10, Arg-1 and CD206 by inhibiting TLR4-related NF-κB and MAPK signaling pathways (11). In addition, *Lactobacillus* spp can regulate Mφ polarization by activating STAT1 and NF-κB p65 (12).

*Bacillus*, a probiotic, is widely used to prevent gastrointestinal disorders and improve animal growth performance (13, 14). Typically, *Bacillus amyloliquefaciens* SC06 (Ba), which is isolated from soil, has a wide range of biological activities (15–19). Our preliminary research showed that Ba could regulate the macrophages phenotype in immune responses. C57BL/6 mice were orally administered Ba and the results showed that only the number of macrophages increased significantly *in vivo*. Additionally, the expression of M1 and M2 macrophages phenotypic genes was altered *in vitro* and Ba induced Raw264.7 cells resulting in polarization to M1 (20). Ba decreased the ratio of *Firmicutes/Bacteroidetes* and increased *Saccharibacteria* phylum (*TM7*) abundance in the gut microbiota of high-fat diet-fed C57BL/6J mice (18). However, it remains unclear what substance secretion by *Bacillus* takes part in *Bacillus*-mediated Mφ polarization and function.

Exosomes are one type of extracellular vesicles derived from the fusion of multivesicular bodies with the plasma membrane to release intraluminal vesicles into the extracellular matrix. Almost all cell types, including intestinal epithelial cells, lymphocytes, nerve cells, mesenchymal stem cells, and tumor cells, are capable of releasing exosomes (21, 22). Exosomes can also act as mediators of signal transmission between cells through specific cell surface proteins, and through their internal structure and composition, to play a role in remote regulation. Exosomes contain proteins, lipids and RNA, as well as abundant miRNAs in total RNA. miRNAs are a class of endogenous single-stranded mature non-coding RNAs of approximately 18-22 nucleotides that can be selectively loaded into exosomes and serve as bridges between signal exchange and transmission between cells (23). Studies have shown that exosomal miRNAs have extremely significant regulatory effects on cell growth, proliferation, differentiation, angiogenesis, and immuno-regulation. Despite this evidence, few studies have investigated probiotic regulated macrophage *via* exosomes. The role of exosomes in the Mφ polarization remains obscure.

The secretory products of intestinal epithelial cells play a key role in intestinal immunity (24). The secretions contain not only cytokines and chemokines, but also a large number of exosomes (25). Exosomes are composed of proteins, lipids and RNA. They can serve as transmitters for transporting molecular substances between cells and participate in exerting a regulatory effect on remotely targeted cells by delivering their content (26). In our previous studies, we demonstrated that Ba can mediate Mφ polarization to M1, enhance phagocytic activity, and regulate intestinal microbiota structure *in vitro* (18, 20). Although ingested bacteria can directly contact and interact with Mφ inside the intestinal tract, most of Mφ can be separated from the bacteria for the intestinal barrier. Therefore, we deduced Ba may regulate the phenotype and function of macrophages inside the intestinal tract by inducing secretion of exosomes from intestinal epithelial cells.

In this study, we isolated exosomes from Ba-induced intestinal porcine epithelial cells lines (IPEC-J2) and investigated their regulation of biological functions of Mφ. We found that exosomes released from IPEC-J2 treated with Ba did not affect Mφ apoptosis. However, they can enter into Mφ, promote phagocytosis, and stimulate Mφ polarization to M1. These findings expand the current knowledge on probiotics-mediated immunity and provide a new understanding of the relationship between probiotics and intestinal immunity.

## Materials and methods

### Reagents

The PKH67 green fluorescent cell linker kit, horseradish peroxidase-conjugated anti-mouse immunoglobulin G, and horseradish peroxidase-conjugated anti-rabbit immunoglobulin G were obtained from Sigma-Aldrich (St. Louis, MO, USA). LPS (*Escherichia coli* 0111: B4) and fluorescein isothiocyanate dextran (FITC-dextran; 40,000 Da) were purchased from Sigma-Aldrich (St. Louis, MO, USA). IFN-γ was purchased from eBioscience (San Diego, CA). Recombinant Porcine IL-4 was obtained from Bio-Techne Co., Ltd. (Minneapolis, MN, USA). CD63, ALIX, and TSG101 were obtained from Abcam (Cambridge, UK). Annexin V-FITC/PI Apoptosis Detection Kit was purchased from Vazyme Biotech Co., Ltd. (Nanjing, China).

### Probiotic preparation and cell culture

The probiotic Ba was isolated from the soil and deposited at the China Center for Type Culture Collection (CCTCC No: M 2012280). Ba was grown in Luria-Bertani medium overnight at 37°C, harvested by centrifugation at 5000 rpm for 10 min, washed completely and suspended in Dulbecco’s phosphate buffered saline (DPBS) at optical densities at 600 nm. The inactivated bacterial precipitate was collected according to a previously described method (19) and used in subsequent experiments.

IPEC-J2 was obtained from the Cell Bank of the Chinese Academy of Sciences, Shanghai, China, and cultured in DMEM-F12 medium (Hyclone, Logan, UT, USA) supplemented with 10% exosome-free fetal bovine serum (FBS), 100 U/mL penicillin (Sigma-Aldrich, St. Louis, MO, USA), and 100 μg/mL streptomycin (Sigma-Aldrich, St. Louis, MO, USA) at 37°C in an incubator with 5% CO_2_. Porcine alveolar macrophage cell line 3D4/21 was donated by the Institute of Animal Husbandry and Veterinary Medicine Zhejiang Province and maintained in cell culture medium (10% FBS, 1% MEM non-essential amino acids, 100 U/mL penicillin, and 100 µg/mL streptomycin in RPMI 1640 medium) at 37°C in 5% CO_2_ atmosphere. 3D4/21 cells were fusiform, with clear outlines and obvious boundaries. It is an effective tool to study the immune characteristics and virus infection mechanism (27, 28).

### Isolation of exosomes

Exosomes were prepared as previously described with modifications (29). Briefly, IPEC-J2 cells grew and reached an appropriate density. After 72 h of incubation with inactivated Ba, the supernatant was collected and treated with DPBS as the control group. The cell culture supernatant was centrifuged at 480 × *g* for 5 min and 2000 × *g* for 10 min to remove dead cells and cell debris. The supernatant was filtered through a 0.22-μm pore filter (EMD Millipore, Billerica, MA, USA). A portion of the filtered supernatant was used for polymerase chain reaction (PCR) to avoid bacterial contamination. The remaining supernatant was subjected to ultracentrifugation at 100,000 × *g* for 70 min, and the precipitate was collected and washed with DPBS by centrifugation at 130,000 × *g* for 1 h. The pellets were then carefully resuspended in DPBS, pooled into a new tube, and centrifuged again. The exosome-enriched fraction was diluted with 100 μL of DPBS and stored at -80°C. All centrifugations were performed at 4°C. The bicinchoninic acid protein assay kit (Pierce, Waltham, MA, USA) was used to determine the protein content of the concentrated exosomes.

### Identification of exosomes

The morphology of the exosomes was imaged using transmission electron microscopy (Tecnai 10) after staining with 5% uranyl acetate. Concentrations and diameters were determined using a ZetaView^®^ Nanoparticle Tracking Analyzer (ZetaView, Particle Metrix, Germany). Moreover, the characterization of exosomes was confirmed by measuring the expression of exosome-specific markers (CD63, ALIX, and TSG101) using western blot analysis.

### Cell cytotoxicity assay

Cell viability was determined using the cell counting kit 8 (CCK-8, Biosharp, China) method as previously reported (19). Briefly, 3D4/21 cells were loaded into a 96-well microplate and incubated with Exo and Ba-Exo at different concentrations (0, 1000, 2000, 3000, 4000, and 5000 particles/cell) for 24 h. The cell supernatants were removed, and 10 µL CCK-8 assay solution was added to each well of a 96-well microplate. After incubation for 1 h. Subsequently, the value Optical density (OD) were measured using a Tecan Spark™ 10M multimode plate reader at OD_450_.

### Cellular uptake analyses

PKH67-labeled exosomes were prepared according to the manufacturer’s instructions using modifications. Briefly, the exosome-enriched fraction from sequential ultracentrifugation was incubated with 500 μL of diluent C solution containing 4 μL of PKH67 for 5 min in the dark, and then bovine serum albumin was added to stop the labeling reaction. After the 100,000 × *g* ultracentrifugation step, supernatants were discarded and pellets were carefully resuspended in DPBS, pooled into a new tube, and centrifuged again. PKH67-labeled exosomes were incubated with 3D4/21 cells at 37°C for 0, 6, 12 and 24 hours. The cells were incubated with 100 ng/mL 4′-6-diamidino-2-phenylindole (DAPI; Invitrogen) at room temperature for 10 min for nuclear staining. Imaging was performed using a Zeiss LSM900 microscope with electron microscopy (Zeiss, Jena, Germany).

### Real-time PCR for expression analysis

3D4/21 cells were pretreated with Exo and Ba-Exo for 24 h, DPBS was used as control. and subsequently stimulated with 150 ng/ml LPS and 50 ng/ml IFN-γ at 37°C for 24 h. Cells were harvested and reverse transcription quantitative PCR (RT-qPCR) analysis of the M1 macrophage marker gene iNOS and M2 macrophage marker gene Arg-1 were performed as previously described (28). The following primers were used: iNOS forward, ACGCTCAGCTCATCCGGTAT, and reverse, CACTTCAGCTCCAGCTCCTG. Arg-1 forward, CCAGTCCATGGAGGTCTGTC, and reverse, GTGTCTTCCCCAGAGATGGA. Exosomal total RNA was reverse-transcribed according to the miScript II Transcriptase Kit protocol (QIAGEN). Relative expression levels were calculated using the 2^-ΔΔCT^ method with U6 snRNA for normalization. The primers used were the following: miR-24-3p, GCGTGGCTCAGTTCAGCAG; miR-27a, CGCGTTCACAGTGGCTAAG; miR-127, GCGTCGGATCCGTCTGAGC; miR-181a, CGAACATTCAACGCTGTCGG; miR-222, GCGAGCTACATCTGGCTACTG; and U6 snRNA, CGTGCTCGCTTCGGCAG. In all cases, the samples were run in triplicate.

### Phagocytosis assay

To assess the phagocytic activity of 3D4/21 cells with exosomes, cells were pretreated with Exo and Ba-Exo for 24 h and then incubated with FITC-dextran at 37°C for 1 h. Cells were fixed with 2% paraformaldehyde at room temperature for 10 min. After washing with DPBS, the cells were incubated with 100 ng/mL DAPI (Invitrogen) at room temperature for 7 min for nucleus staining. Imaging was performed using a laser scanning confocal microscope Zeiss LSM900 (Zeiss, Germany). Furthermore, fluorescence signals were detected using a BD FACSFortessa multicolor flow cytometer (BD Biosciences, USA) at 488 nm. At least 10,000 events were collected from the cell gates. The data were then analyzed using FlowJo software (BD Biosciences).

### Apoptosis assay

To assess the effect of exosomes on the apoptosis of 3D4/21 cells, cells were pretreated with Exo and Ba-Exo for 24 h, and the apoptosis rate was detected by flow cytometry in accordance with the manufacturer’s instructions. Cells were collected and suspended in permeabilization buffer (2% FBS and 0.2% Tween-20 in DPBS) for 10 min at room temperature. After washing with cold DPBS, the cells were suspended in 100 μL of 1× binding buffer and mixed by gentle back-pipetting until a single-cell suspension was obtained. The cells were incubated with 5 μL of Annexin V-FITC and 5 μL of propidium iodide staining solution for 5 min at room temperature, protected from light. After washing with cold DPBS, the apoptosis rate was determined using a BD FACSFortessa multicolor flow cytometer (BD Biosciences, USA) at 488 and 561 nm. At least 10,000 events were collected from the cell gates. The data were then analyzed using FlowJo software (BD Biosciences).

### Statistical analysis

All data are expressed as the mean ± standard deviation of three independent experiments. Statistical evaluation of differences between means of experimental groups was performed using two-tailed Student’s t-test and one-way ANOVA analysis of variance using SPSS 20.0 statistical software (SPSS Inc., Chicago, IL, USA). Statistical significance was considered at *p<0.05 or **p<0.01.

## Results

### Characterization of Ba-Exo and Exo

**Figure 1:**
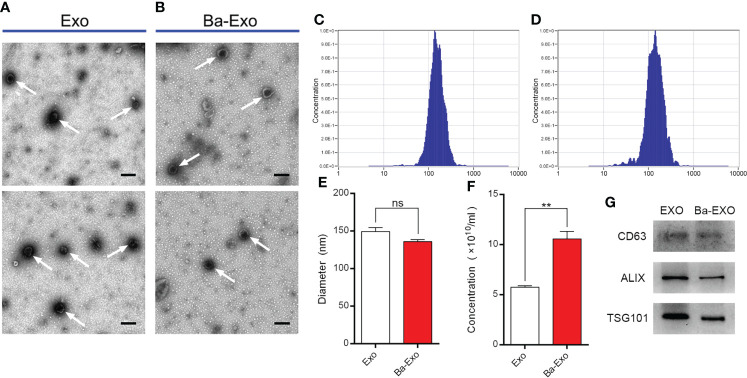
Characterization of exosomes (Ba-Exo and Exo) from IPEC-J2 with or without Ba-treatment. **(A, B)**, Representative images of Ba-Exo and Exo by transmission electron microscopy. Arrowheads indicate the cup-shaped vesicles. Scale bar: 200 nm. **(C, D)**, The size distribution of exosomes Exo and Ba-Exo by nanoparticle tracking analysis. **(E)**, Statistical analysis of the diameters between exosomes Exo and Ba-Exo. **(F)**, Statistical analysis of the concentration between exosomes Exo and Ba-Exo. **(G)**, Western blot analysis of the typical exosomes-associated proteins. Mean ± SD of three independent experiments are shown. **p < 0.01, ns, not significant. IPEC-J2, intestinal porcine epithelial cell lines; SD, standard deviation.

We obtained two exosomes (Exo and Ba-Exo) secreted by IPEC-J2 cells by ultracentrifugation, of which Exo was derived from untreated IPEC-J2 and Ba-Exo was derived from Ba-induced IPEC-J2. Exo and Ba-Exo have typical cup-shaped and double-layered phospholipid membrane structures (**Figures 1A, B**). Nanoparticle tracking analysis showed that Exo and Ba-Exo had a tighter peak of comparable sizes (130-150 nm) (**Figures 1C-E**). Moreover, the secretion of exosomes in the Ba-Exo group increased significantly (**Figure 1F**). Western blotting showed that the typical exosome-associated proteins CD63 ALIX and TSG101 were present on their surfaces (**Figure 1G**). These results demonstrate that Exo and Ba-Exo display specific characteristics of exosomes, which is consistent with previous reports (30).

### Ba-Exo and Exo can transfer into 3D4/21 Cells effectively

**Figure 2:**
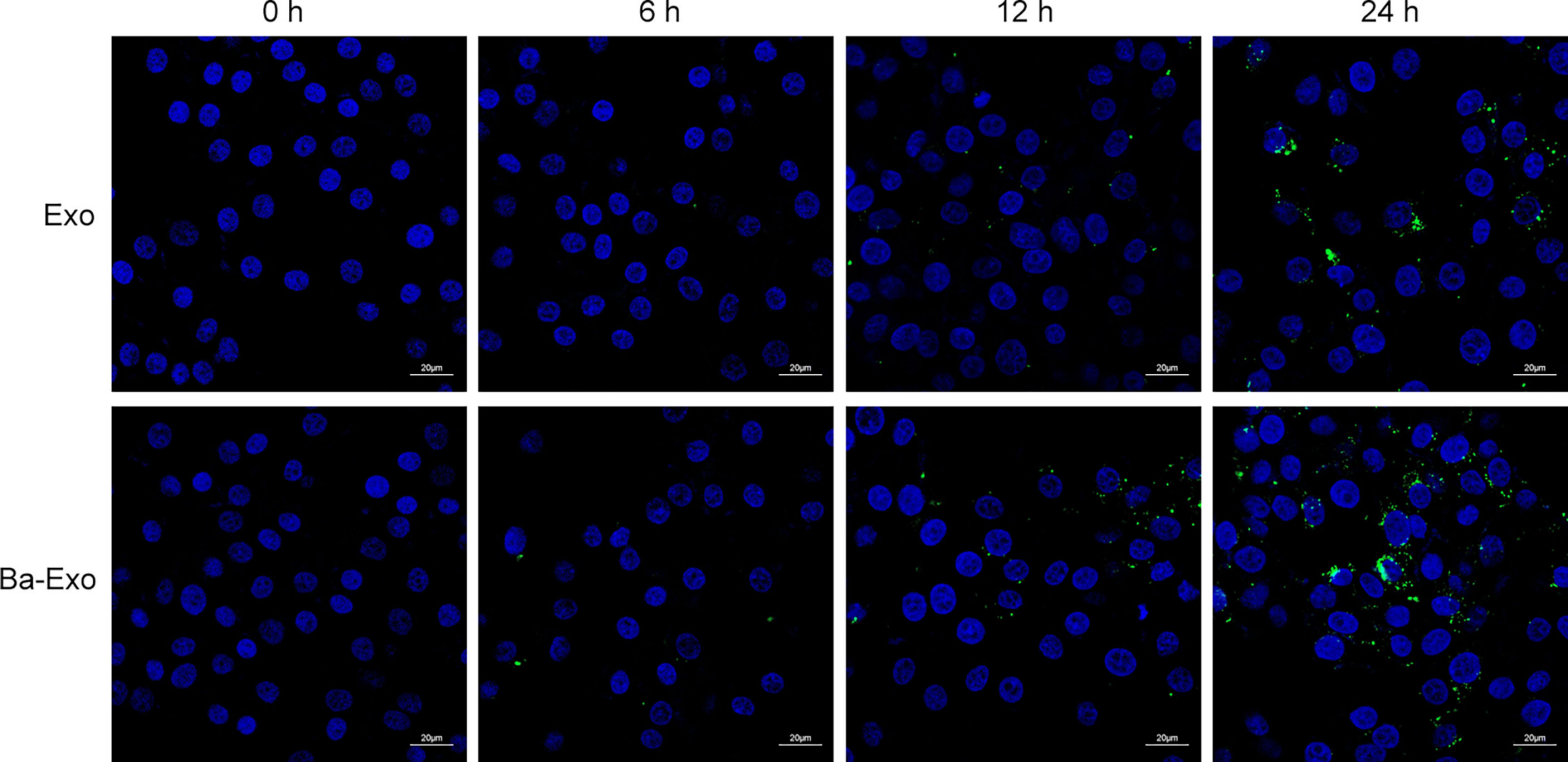
Time-course analysis of Exo and Ba-Exo uptake by Mφ (3D4/21). Representative images of Exo and Ba-Exo uptake by 3D4/21 after PKH67 green fluorescent dye labeling.

We determined whether IPEC-J2 can secrete exosomes, which are then transported into the target cells. The green fluorescent PHK67 labeled Ba-Exo and Exo separately, which were then incubated with 3D4/21 cells. The results showed that Ba-Exo and Exo could penetrate and enter 3D4/21 cells effectively after 24 h (**Figure 2**). This result indicated that Ba-Exo and Exo can enter Mφ effectively and provide the basis for subsequent functional research in target cells.

**Figure 3:**
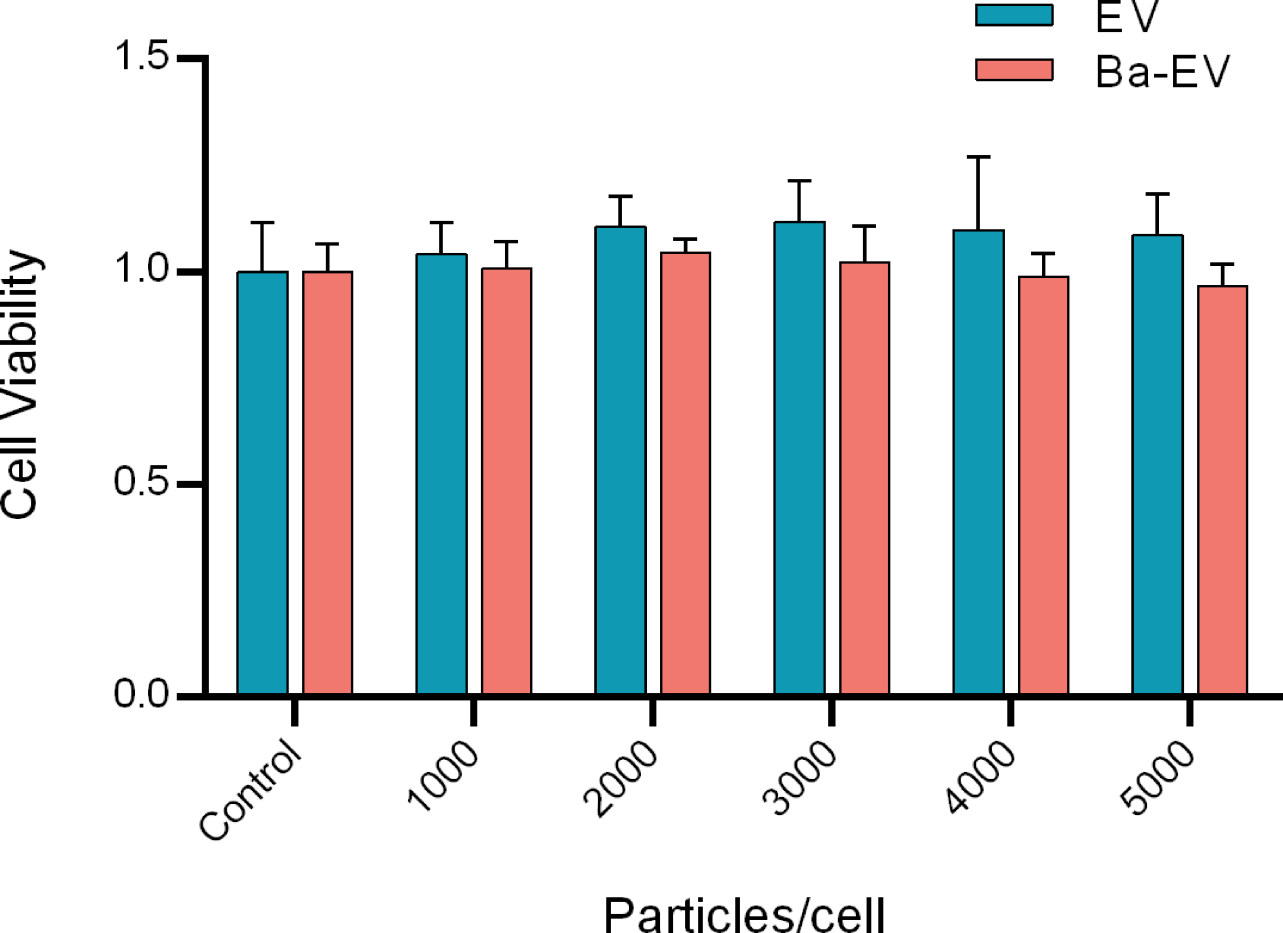
Toxicity assay of different doses of Exo and Ba-Exo on 3D4/21 cells. 3D4/21 cells were treated with different dosages (0, 1000, 2000, 3000, 4000, and 5000 particles/cell) for 24 h. Dulbecco’s phosphate buffered saline served as the negative control. Cell viability was determined by the cell counting kit 8 method.

To determine the optimal dose of exosomes for subsequent studies, 3D4/21 cells were treated with different dosages (0-5000 particles/ml) for 24 h. Finally, the toxicity of the cells was detected using the CCK-8 assay. The results showed that the ratio of the number of exosomes added to 3D4/21 cells reached 5000 particles/cell, and cell viability was not affected (**Figure 3**). Therefore, these concentrations and incubation times were the optimal choices for subsequent experiments.

### Ba-Exo promote phagocytic activity of 3D4/21 cells

**Figure 4:**
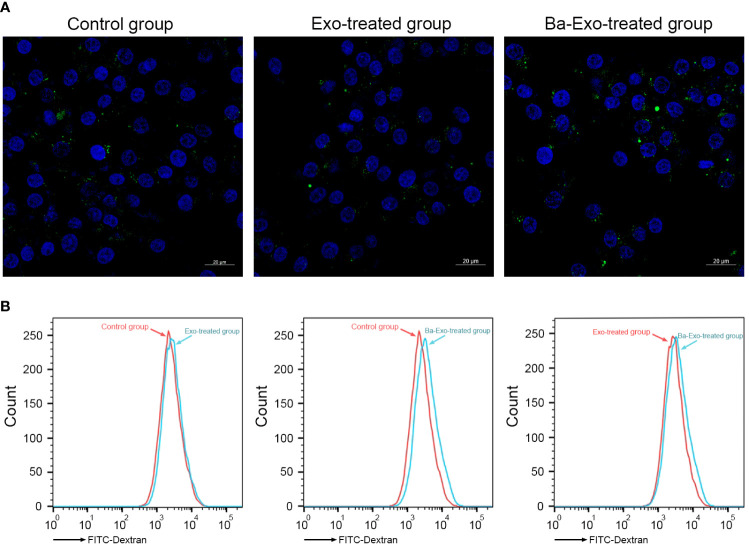
Ba-Exo promotes Mφ phagocytosis. The phagocytosis of 3D4/21 cells were measured by the quantitative uptake of fluorescein isothiocyanate dextran (FITC-dextran) after Ba-Exo and Exo pretreatment. **(A)**, The quantitative uptake of FITC-dextran was detected by laser-scanning microscope. Representative images were captured under a laser scanning confocal microscope (Zeiss LSM900). **(B)**, The quantitative uptake of FITC-dextran was detected by BD FACSCanto™ flow cytometer.

Cell phagocytosis plays an important role in Mφ phagocytosis of antigens and elimination of pathogen infection. To investigate the effects of exosomes on Mφ phagocytosis, we used green fluorescent FITC-dextran to incubate 3D4/21 cells and detect their phagocytic capacity. The results showed that the quantity and fluorescence intensity of green fluorescent FITC-dextran in the Ba-Exo group were significantly higher than those in the control and Exo groups (**Figure 4A**). Flow cytometry analysis also confirmed that phagocytosis of 3D4/21 cells in the Ba-Exo group was upregulated compared to that in the Exo and control groups (**Figure 4B**). These observations suggest that Ba-Exo can promote the phagocytic activity of 3D4/21 cells.

### Ba-Exo and Exo have no effect on Mφ apoptosis

**Figure 5:**
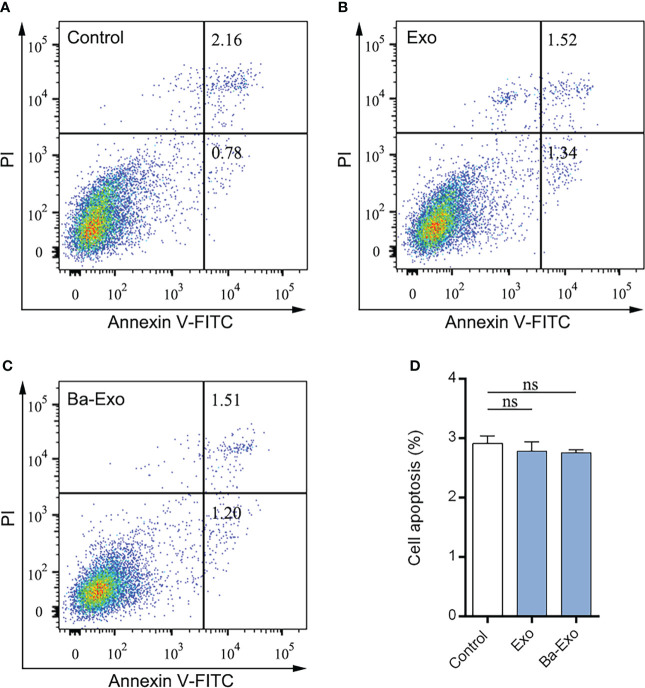
Evaluation of apoptosis levels were determined. Dulbecco’s phosphate buffered saline–treated 3D4/21 served as a negative control **(A)**. The 3D4/21 apoptosis levels were measured by flow cytometry after Exo **(B)** and Ba-Exo **(C)** pretreatment. **(D)**, Quantification of apoptotic cells in 3D4/21. Mean ± SD of three independent experiments are shown. SD, standard deviation; ns, not significant.

Apoptosis is critical for various developmental processes. To assess the effects of exosomes on 3D4/21 cell phagocytosis, we performed apoptosis assays using flow cytometry to detect changes in Mφ apoptosis. The results showed that Ba-Exo had no effect on Mφ apoptosis in the Exo group (p<0.05) (**Figure 5**).

### Ba-Exo promote 3D4/21 cells polarization

**Figure 6:**
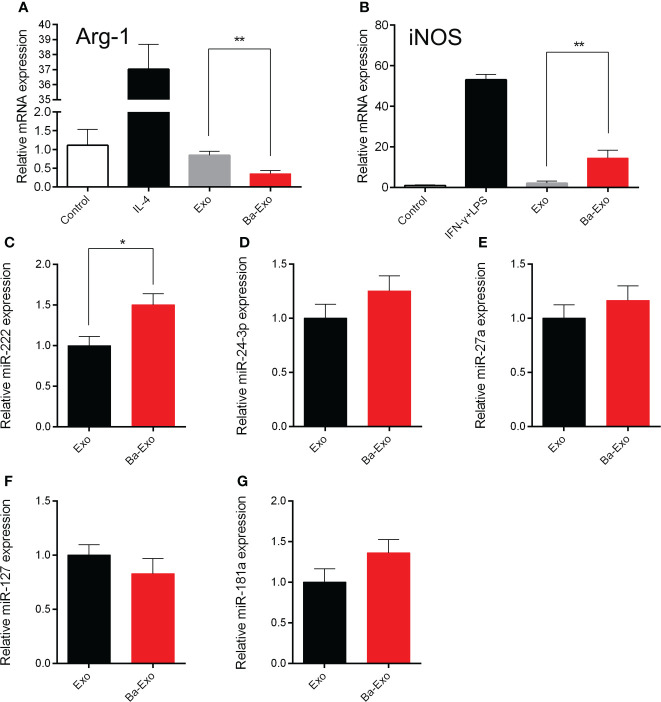
Ba-Exo can promote Mφ polarization to M1. Ba-Exo can decrease the Arg-1 mRNA expression **(A)** and improve the iNOS mRNA expression **(B)**. The reactions were run in combination with the endogenous β-actin control. The total RNA was extracted and transcribed from Ba-Exo and Exo group. Validation of miR-222 **(C)**, miR-24-3p **(D)**, miR-27a **(E)**, miR-127 **(F)**, miR-181a **(G)** expression in Ba-Exo and Exo by quantitative polymerase chain reaction assay. The relative expression levels were calculated using the 2^-ΔΔCT^ method with U6 snRNA for normalization. Mean ± SD of three independent experiments are shown. *p < 0.05, **p < 0.01. Arg1, arginase 1; iNOS, inducible nitric oxide synthase; SD, standard deviation.

Macrophages can differentiate into M1 and M2 macrophages (31). M1 macrophages are capable of clearing intracellular pathogens and pro-inflammatory responses, resulting in a Th1-type immune response. However, M2 macrophages have immunomodulatory and strong tissue repair capabilities (32, 33). Previous studies have shown that Ba promotes Mφ polarization to M1 and enhances its phagocytic activity *in vitro* (20). To evaluate whether Ba-Exo derived from IPEC-J2 cells treated with Ba regulates Mφ polarization, we used fluorescent RT-PCR to detect the expression of polarization-related genes in 3D4/21 cells after exosome stimulation. The results showed that Ba-Exo enhanced the expression of iNOS mRNA in 3D4/21 cells (p<0.05), while inhibiting the expression of Arg-1 mRNA (p<0.05) (**Figures 6A, B**). These results indicated that Ba-Exo promote macrophage polarization to M1 cells.

### Differential miRNA expression analysis between Ba-Exo and Exo

Previous studies have confirmed that miRNAs can regulate Mφ polarization (34, 35). To explore the differences in regulation of Mφ polarization between Ba-Exo and Exo, we chose these Mφ polarization-associated miRNAs (miR-222, miR-24-3p, miR-27a, miR-127, miR-181a) to performed RT-qPCR assay between Ba-Exo and Exo group (**Figures 6C–G**). Among these miRNAs, we found that miR-222 expression was significantly higher in the Ba-Exo group than in the Exo group (p<0.05) (**Figure 6C**). Furthermore, we used the corresponding miRNA inhibitors to inhibit the corresponding genes, and then tested whether Mφ polarize into M1. The result showed that Mφ could still polarize to M1 after these miRNA (miR-222, miR-24-3p, miR-127, miR-27a and miR-181a) were inhibited respectively (p>0.05) (**Supplementary Figure S1**).

## Discussion

**Figure 7:**
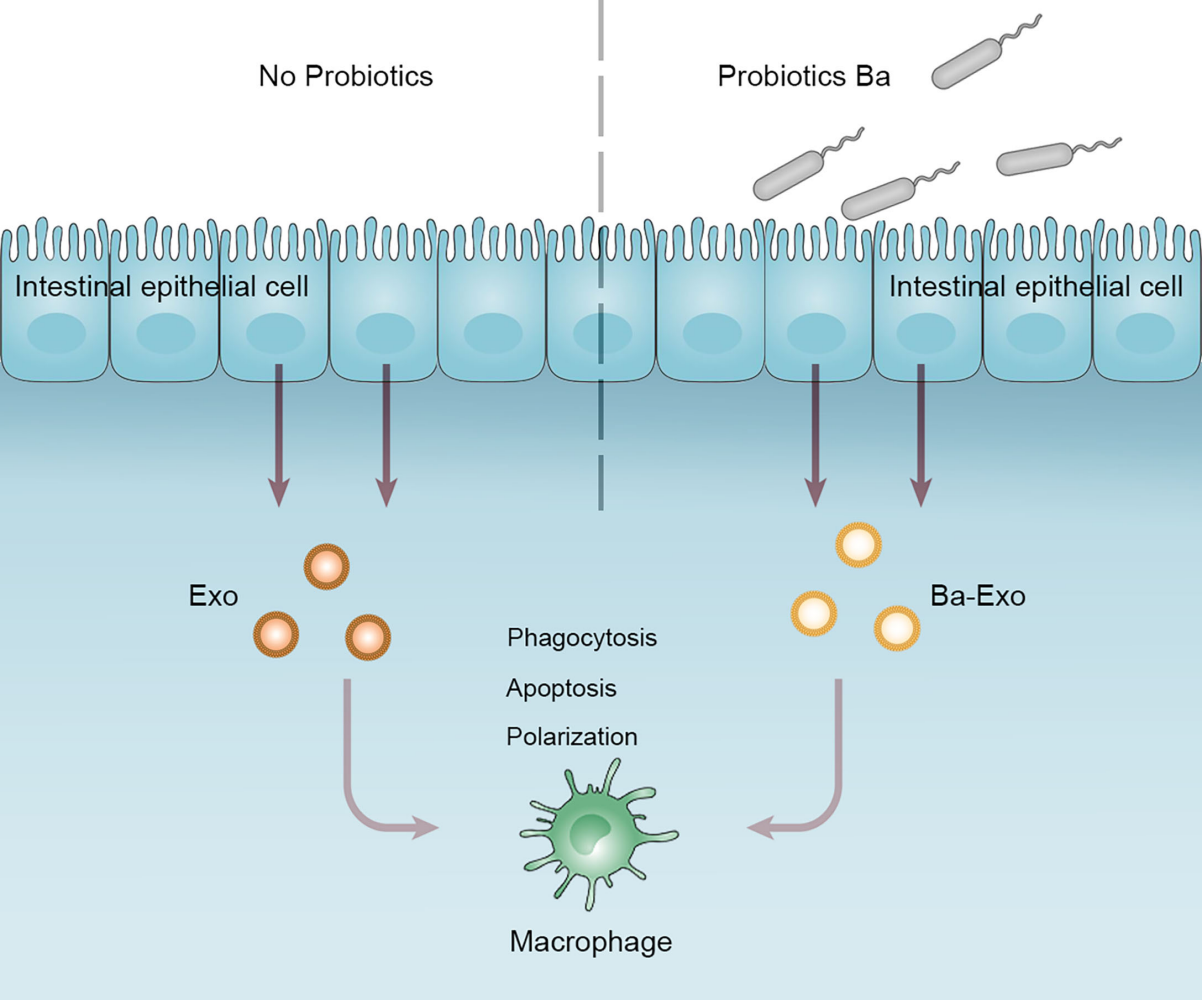
Working model depicting a proposed role of Ba-Exo modulating Mφ function. Exosomes (Ba-Exo) released from intestinal porcine epithelial cells treated with Ba had no effect on Mφ apoptosis. However, they can enter Mφ, promote phagocytosis of Mφ, and stimulate Mφ polarization to M1. Ba, *Bacillus amyloliquefaciens* SC06; Exo, exosomes derived from untreated IPEC-J2; Ba-Exo, exosomes secreted from Ba-induced IPEC-J2.

We collected exosomes (Ba-Exo) derived from intestinal epithelial cells treated with Ba and investigated the regulation of Mφ phagocytosis, apoptosis, and polarization. We found that Ba-Exo could enter Mφ, promote phagocytosis of Mφ, and stimulate Mφ polarization to M1. This provides a novel perspective to reveal the relationship between probiotics and Mφ, regulate intestinal immunity, and maintain intestinal homeostasis (**Figure 7**).

In recent years, studies on the regulation of intestinal immunity by exosomes have become a trending research topic. Brown et al. found that lymphatic endothelial cells induce dendritic cell migration and maturation through exosomes and participate in the immune response of the body. They found that exosomes carry chemokines such as CX3CL1 and interact with the receptor molecule GPCR on the dendritic cell surface (36). Similarly, we also found that exosomes secreted by intestinal epithelial cells can promote the migration of Mφ (data not shown); however, they explored the mechanism of Mφ migration from the perspective of exosomal small RNA. Zhuang et al. found that miR-103 in serum and liver cancer cell culture supernatants of patients with hepatocellular carcinoma can increase endothelial cell permeability and inhibit the expression of endothelial cells VE-Cad, p120, and ZO-1, as well as p120-E-Cad signaling, thereby attenuating endothelial cell junctions and promoting cancer cell migration (37). Our experiments also demonstrated that Ba-Exo promoted Mφ migration. However, the specific mechanisms require further investigation.

miRNAs also play an important role in Mφ polarization. Various miRNAs have been found to regulate Mφ polarization by targeting Mφ polarization-related proteins. Graft et al. found that there are eight miRNAs with significant expression differences in human M1 and M2 Mφ (miR-155, miR-125a, miR-132, miR-27a, miR-193b, miR-29b, miR-222, and miR-26a). Overexpression of miR-29b and miR-125a in human monocyte cell lines can target tumor necrosis factor-inducible protein 3 (TNFAIP3). TNFAIP3 inhibits the NF-κB signaling pathway, and miR-29b and miR-125a increase the expression of M1 Mφ markers by inhibiting TNFAIP3 expression (34). Zhang et al. also found that 109 miRNAs were expressed at different levels in M1 and M2 in murine bone marrow-derived Mφ, and differentially expressed miRNAs were screened using quantitative PCR: miR-181a, miR-155, miR-204, miR-451, miR-125, miR-146a, miR-143, and miR-145 (38). Liu et al. found that M1 expressed more miR-125a than M2, and overexpression of miR-125a reduced LPS-mediated expression of M1-related phenotype molecules but promoted IL-4-mediated induction of M2-type giants. Knockdown of miR-125a promotes M1-type polarization and reduces IL-4-mediated expression of M2-related phenotypic molecules (39). We conclude that Mφ in the intestine maintain colonization and balance of intestinal flora by secreting cytokines and microvesicles, including exosomes. Mφ polarization may limit the number of pathogenic bacteria, which is beneficial for the maintenance of probiotics, and balance the intestinal flora (32).

In addition, the polarization of intestinal Mφ is also an early warning mechanism that helps the body resist antigen invasion and efficiently eliminates pathogenic microorganisms. We performed a RT-qPCR assay of the polarization-related mall RNAs (miR-222, miR-24-3p, miR-27a, miR-127, and miR-181a) between the Ba-Exo and Exo groups. Among these miRNAs, we found that miR-222 expression was significantly higher in the Ba-Exo group than in the Exo group.

Probiotic Ba played a key role in protecting macrophages against *E. coli* infection. It can improve the expression of Beclin1 and Atg5-Atg12-Atg16 complex to induced the autophagy in RAW264.7 cells (19). Oxidative stress is thought to be associated with the gastrointestinal disorders. Probiotic Ba alleviate the oxidative stress of IPEC-1 *via* modulating Nrf2/Keap1 (40). Since oxidative stress is a common phenomenon in obesity, Ba can also prevent obesity by regulating the antioxidant capacity and gut microbiota of hosts (18, 41). Furthermore, Ba could shape the intestinal microbial composition, change metabolites, and regulate bile acid metabolism, which eventually alleviate the obesity of male ob/ob mice (15). Moreover, Ba can induce AKT-FOXO-mediated autophagy to alleviate oxidative stress-induced apoptosis and cell damage in IPEC-J2 cells (17). These findings may aid in the application of probiotic Ba in food to improve the host’s immunology and health.

The relationship between the microbiome and Mφ polarization is still obscure. Mφ polarization can regulate immunity by activating signaling pathways and secretion of related cytokines (42). Studies have shown that microbiome interactions with Mφ also improve host immunity (43). Probiotic VSL#3 ameliorated renal ischemia-reperfusion injury by modulating the phenotype of Mφ through the IL-10/GSK-3β/PTEN signaling pathway (44). *Lactobacillus* strains enhance phagocytosis and the bactericidal activity of Mφ through NF-κB- and TLR2-dependent signaling pathways (45). Ba pretreatment attenuated the activation of JNK in RAW264.7 cells during *E. coli* infection (19). Therefore, we speculate that probiotics may activate Mφ to improve host immunity by regulating the gut microbiome and activating other signaling pathways.

## Conclusion

The present study provides substantial evidence confirming that probiotic, Ba, induces intestinal epithelial cells to produce Ba-Exo and regulate Mφ biological functions. This modulated mechanism plays an essential role in the host gastrointestinal tract to maintain or improve health and reduce the risk of disease. Given that probiotics play pivotal roles in gut immunity, we also revealed that Ba-Exo has important effects on Mφ and provides a reference for food and clinical applications in the future.

## Data availability statement

The original contributions presented in the study are included in the article/**Supplementary material**. Further inquiries can be directed to the corresponding authors.

## Author contributions

All the authors reviewed and approved the final version of the manuscript and agreed to be accountable for the content of the work. XX, GM, ZlZ, and WL conceived and designed the experiments; RL, XZ, LC, TZ, YH, and YW performed the experiments; RL, XZ, XX, YH, and ZsZ, analyzed the data; RL, XX, and ZsZ made the figures; XX, GM, ZlZ, and RL wrote the paper.

## Funding

This study is supported by National Natural Science Foundation of China (No.31702144, 31672460 and 31472128), Zhejiang Province Basic public welfare research project (LGF21H250002 and LGN21D060001), Chinese Traditional Medicine Science and Technology Projects of Zhejiang Province (2021ZB002, 2022ZB002), Health Bureau of Zhejiang Province (2019RC092, 2022KY463, and 2020KY394). Dr. Gen-Xiang Mao was an Irma and Paul Milstein Program for Senior Health fellow supported by the MMAAP Foundation (https://www.mmaapf.org/). The authors thank all workers of the study for their participation.

## Conflict of interest

The authors declare that the research was conducted in the absence of any commercial or financial relationships that could be construed as a potential conflict of interest.

## Publisher’s note

All claims expressed in this article are solely those of the authors and do not necessarily represent those of their affiliated organizations, or those of the publisher, the editors and the reviewers. Any product that may be evaluated in this article, or claim that may be made by its manufacturer, is not guaranteed or endorsed by the publisher.
