# Supplementary material for: Characterization of exosomes derived from IPEC-J2 treated with probiotic Bacillus amyloliquefaciens SC06 and its regulation of macrophage functions
Source: Front Immunol. 2022 Nov 9;13:1033471. doi: 10.3389/fimmu.2022.1033471 (PMC9682075; doi:10.3389/fimmu.2022.1033471)
Supplement: Supplementary file 1 [file Image_1.pdf]

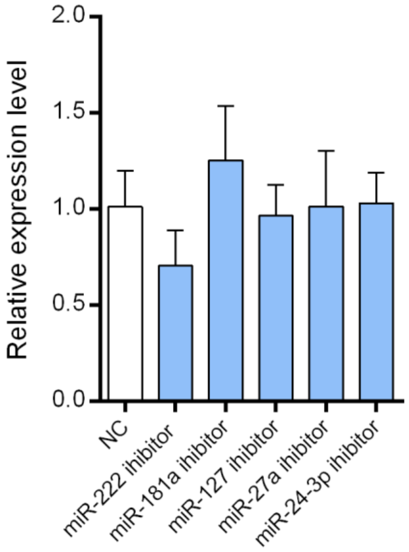

Supplementary Figure 1. Effects of miRNA inhibitor on M1 macrophage marker iNOS gene expression. 3D4/21 cells were pretreated with miR-222, miR-24-3p, miR-127, miR-27a and miR-181a respectively, and subsequently stimulated with LPS and IFN-γ at 37 ℃ for 24 h. Cells were harvested and quantitative real-time PCR analysis of iNOS.
